# Supplementary material for: Cross-sectional study to describe allergic rhinitis flare-ups and associated airways phenotype in house dust mite sensitization
Source: PLoS One. 2023 Mar 23;18(3):e0283246. doi: 10.1371/journal.pone.0283246 (PMC10035855; doi:10.1371/journal.pone.0283246)
Supplement: S1 File — (DOCX) [file pone.0283246.s001.docx]

**S1 Underlying data set**

**Staffing Justification:**

The main objective of the study is to identify and describe episodes of exacerbations of respiratory allergy (exacerbations of rhinitis and/or asthma) within 12 months prior to the study in patients who are offered a mite AIT-SL due to severe allergic rhinitis. T

he calculation of the number of subjects required is based on the rate of events in allergic rhinitis more common than in asthma. We used data from the study Antares published by Demoly et al in 2016 [1] in rhinitis, the use of data on asthma would have led to too many effects (cf. Gayraud et al [2], study Adara, 2013).

An expected frequency of exacerbation of allergic rhinitis of the order of 10 to 20% [1] (on the Significant modification criterion of usual treatments) for the population Overall, requires an effective between 1 768 and 2 079 patients:

**
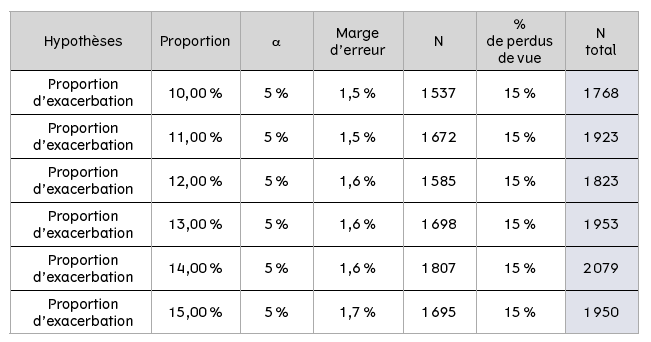
**

Based on an adults/adolescents/children distribution estimated from the Antares study [3], by 60%, 20% and 20% respectively, the effects to be included would be approximately 1,200 adults, 400 adolescents and 400 children. With these effects, the accuracy of measuring the rate of exacerbation will be of the order of 1.8 to 2.2% for the adult population, from 3.2% to 3.8% for adolescent populations and infantile.

Taking into account a rate of missing or unanalysable data of approximately 15%, a total effective of about 2 000 patients, should make it possible to have sufficient details to describe the frequency of exacerbation and its interval of 95% confidence.

To achieve this goal, with the average assumption of 10 to 12 patients per physician, the participation of about 170 to 200 physicians is required.

[1] Demoly P, Emminger W, Rehm D, Backer V, Tommerup L, Kleine-Tebbe J. Eff ective treatment of house dust mite-induced allergic rhinitis with 2 doses of the SQ HDM SLIT-tablet : Results from a randomized, double-blind, placebo-controlled phase III trial. J Allergy Clin Immunol 2016; 137(2): 444-451.

[2] Gayraud, J., Refabert, L., & Chartier, A. (2013). Infl uence of asthma on specifi c immunotherapy practices in patients with house dust mite-induced allergy. An observational study AdArA. Revue française d’allergologie; 53(5): 458-467.

[3] Demoly P, Broué-Chabbert A, Wessel F, Chartier A. Severity and disease control before house dust mite immunotherapy initiation : ANTARES a French observational survey. Allergy Asthma Clin Immunol 2016; 12:13.

**Results:**

| Table 22 Age of diagnosis of HDM rhinitis (years) |
| --- |

|  | | **Total N=1701** |
| --- | --- | --- |
| **Age of diagnosis of HDM rhinitis (years)** | N | 1684 |
|  | Missing | 17 |
|  | Mean ± ET | 3.2 ± 5.9 |
|  | Median | 1 |
|  | Q1 ; Q3 | 0 ; 3 |
|  | Min. ; Max. | -1 ; 57 |
|  | | |

|  | | **from 5 to 11 years N=513** | **12-17 years N=298** | **Adults N=875** |
| --- | --- | --- | --- | --- |
| **Age of diagnosis of HDM rhinitis (years)** | N | 511 | 297 | 865 |
|  | Missing | 2 | 1 | 10 |
|  | Mean ± ET | 1.3 ± 1.7 | 2.6 ± 3.3 | 4.5 ± 7.6 |
|  | Median | 1 | 1 | 1 |
|  | Q1 ; Q3 | 0 ; 2 | 0 ; 4 | 0 ; 6 |
|  | Min. ; Max. | 0 ; 11 | 0 ; 17 | -1 ; 57 |
|  | | | | |

| Table 33 Symptoms |
| --- |

|  | | **Total N=1701** |
| --- | --- | --- |
| **Sneezing** | N | 1701 |
|  | No | 61 (3.6%) |
|  | Yes | 1640 (96.4%) |
|  | | |
| **Rhinorrhea** | N | 1701 |
|  | No | 54 (3.2%) |
|  | Yes | 1647 (96.8%) |
|  | | |
| **Nasal obstruction** | N | 1701 |
|  | No | 117 (6.9%) |
|  | Yes | 1584 (93.1%) |
|  | | |
| **Nasal pruritus** | N | 1701 |
|  | No | 325 (19.1%) |
|  | Yes | 1376 (80.9%) |
|  | | |
| **Loss of smell** | N | 1701 |
|  | No | 983 (57.8%) |
|  | Yes | 718 (42.2%) |
|  | | |
| **Ocular pruritus** | N | 1701 |
|  | No | 680 (40.0%) |
|  | Yes | 1021 (60.0%) |
|  | | |
| **Redness and tearing** | N | 1701 |
|  | No | 817 (48.0%) |
|  | Yes | 884 (52.0%) |
|  | | |

**Table I:**

| Table 11 Sex |
| --- |

|  | | **Total N=1701** |  |  |
| --- | --- | --- | --- | --- |
| **Sex** | N | 1694 |  |  |
|  | Missing | 7 |  |  |
|  | M | 873 (51.5%) |  |  |
|  | F | 821 (48.5%) |  |  |
|  | | |  |  |
|  | | **from 5 to 11 years N=513** | **12-17 years N=298** | **Adults N=875** |
| **Sex** | N | 513 | 297 | 875 |
|  | Missing | 0 | 1 | 0 |
|  | M | 330 (64.3%) | 168 (56.6%) | 370 (42.3%) |
|  | F | 183 (35.7%) | 129 (43.4%) | 505 (57.7%) |
|  | | | | |

| Table 12 Age (ans) |
| --- |

|  | | **Total N=1701** |
| --- | --- | --- |
| **Age** | N | 1686 |
|  | Missing | 15 |
|  | Mean ± ET | 23.0 ± 15.1 |
|  | Median | 18 |
|  | Q1 ; Q3 | 10 ; 33 |
|  | Min. ; Max. | 5 ; 88 |
|  | | |

|  | | **from 5 to 11 years N=513** | **12-17 years N=298** | **Adults N=875** |
| --- | --- | --- | --- | --- |
| **Age** | N | 513 | 298 | 875 |
|  | Missing | 0 | 0 | 0 |
|  | Mean ± ET | 8.4 ± 1.9 | 14.2 ± 1.7 | 34.6 ± 12.4 |
|  | Median | 9 | 14 | 33 |
|  | Q1 ; Q3 | 7 ; 10 | 13 ; 15 | 25 ; 41 |
|  | Min. ; Max. | 5 ; 11 | 12 ; 17 | 18 ; 88 |
|  | | | | |

| Table 13 Smoking, Use of cannabis |
| --- |

|  | | **Total N=1701** |
| --- | --- | --- |
| **Smoking** | N | 1690 |
|  | Missing | 11 |
|  | No | 1571 (93.0%) |
|  | Yes | 119 (7.0%) |
|  | | |
| **Use of cannabis** | N | 1685 |
|  | Missing | 15 |
|  | No | 1672 (99.2%) |
|  | Yes | 13 (0.8%) |
|  | | |

|  | | **from 5 to 11 years N=513** | **12-17 years N=298** | **Adults N=875** |
| --- | --- | --- | --- | --- |
| **Smoking** | N | 512 | 296 | 872 |
|  | Missing | 1 | 2 | 3 |
|  | No | 504 (98.4%) | 286 (96.6%) | 772 (88.5%) |
|  | Yes | 8 (1.6%) | 10 (3.4%) | 100 (11.5%) |
|  | | | | |
| **Use of cannabis** | N | 512 | 293 | 870 |
|  | Missing | 1 | 5 | 4 |
|  | No | 512 (100.0%) | 293 (100.0%) | 858 (98.6%) |
|  | Yes | 0 (0.0%) | 0 (0.0%) | 12 (1.4%) |
|  | | | | |

| Table 27 Polysensibilisation |
| --- |

|  | | **Total N=1701** |
| --- | --- | --- |
| **Polysensitisation ? (Y/N)** | N | 1679 |
|  | Missing | 22 |
|  | No | 777 (46.3%) |
|  | Yes | 902 (53.7%) |
|  | | |

|  | | **from 5 to 11 years N=513** | **12-17 years N=298** | **Adults N=875** |
| --- | --- | --- | --- | --- |
| **Polysensitisation (Y/N)** | N | 511 | 294 | 863 |
|  | Missing | 2 | 4 | 12 |
|  | No | 270 (52.8%) | 138 (46.9%) | 365 (42.3%) |
|  | Yes | 241 (47.2%) | 156 (53.1%) | 498 (57.7%) |
|  | | | | |

| Table 14 Allergic pathologies |
| --- |
|  |

|  | | **Total N=1701** |
| --- | --- | --- |
| **Conjunctivitis** | N | 1701 |
|  | No | 747 (43.9%) |
|  | Yes | 954 (56.1%) |
|  | | |
| **Sinusitis** | N | 1701 |
|  | No | 1119 (65.8%) |
|  | Yes | 582 (34.2%) |
|  | | |
| **Atopic eczema** | N | 1701 |
|  | No | 1347 (79.2%) |
|  | Yes | 354 (20.8%) |
|  | | |
| **Food allergy** | N | 1701 |
|  | No | 1557 (91.5%) |
|  | Yes | 144 (8.5%) |
|  | | |
| **Allergic urticaria** | N | 1701 |
|  | No | 1571 (92.4%) |
|  | Yes | 130 (7.6%) |
|  | | |

|  | | **from 5 to 11 years N=513** | **12-17 years N=298** | **Adults N=875** |
| --- | --- | --- | --- | --- |
| **Conjunctivitis** | N | 513 | 298 | 875 |
|  | No | 264 (51.5%) | 135 (45.3%) | 346 (39.5%) |
|  | Yes | 249 (48.5%) | 163 (54.7%) | 529 (60.5%) |
|  | | | | |
| **Sinusitis** | N | 513 | 298 | 875 |
|  | No | 405 (78.9%) | 216 (72.5%) | 493 (56.3%) |
|  | Yes | 108 (21.1%) | 82 (27.5%) | 382 (43.7%) |
|  | | | | |
| **Atopic eczema** | N | 513 | 298 | 875 |
|  | No | 381 (74.3%) | 236 (79.2%) | 720 (82.3%) |
|  | Yes | 132 (25.7%) | 62 (20.8%) | 155 (17.7%) |
|  | | | | |
| **Food allergy** | N | 513 | 298 | 875 |
|  | No | 473 (92.2%) | 270 (90.6%) | 803 (91.8%) |
|  | Yes | 40 (7.8%) | 28 (9.4%) | 72 (8.2%) |
|  | | | | |
| **Allergic urticaria** | N | 513 | 298 | 875 |
|  | No | 477 (93.0%) | 278 (93.3%) | 805 (92.0%) |
|  | Yes | 36 (7.0%) | 20 (6.7%) | 70 (8.0%) |
|  | | | | |

| Table 41 ARIA  classification |
| --- |

|  | | **Total N=1701** |
| --- | --- | --- |
| **ARIA classification** | N | 1599 |
|  | Missing | 102 |
|  | Mild intermittent rhinitis | 139 (8.7%) |
|  | Severe intermittent rhinitis | 33 (2.1%) |
|  | Mild persistent rhinitis | 293 (18.3%) |
|  | Severe persistent rhinitis | 1134 (70.9%) |
|  | | |

|  | | **from 5 to 11 years N=513** | **12-17 years N=298** | **Adults N=875** |
| --- | --- | --- | --- | --- |
| **ARIA classification** | N | 484 | 277 | 823 |
|  | Missing | 29 | 21 | 52 |
|  | Mild intermittent rhinitis | 56 (11.6%) | 25 (9.0%) | 57 (6.9%) |
|  | Severe intermittent rhinitis | 8 (1.7%) | 2 (0.7%) | 23 (2.8%) |
|  | Mild persistent rhinitis | 114 (23.6%) | 46 (16.6%) | 130 (15.8%) |
|  | Severe persistent rhinitis | 306 (63.2%) | 204 (73.6%) | 613 (74.5%) |
|  | | | | |

| Table 40 Asthmatic patient |
| --- |

|  | | **Total N=1701** |
| --- | --- | --- |
| **Asthmatic patient (Y/N)** | N | 1661 |
|  | Missing | 40 |
|  | No | 1089 (65.6%) |
|  | Yes | 572 (34.4%) |
|  | | |

|  | | **from 5 to 11 years N=513** | **12-17 years N=298** | **Adults N=875** |
| --- | --- | --- | --- | --- |
| **Asthmatic patient (Y/N)** | N | 505 | 288 | 854 |
|  | Missing | 8 | 10 | 21 |
|  | No | 290 (57.4%) | 182 (63.2%) | 606 (71.0%) |
|  | Yes | 215 (42.6%) | 106 (36.8%) | 248 (29.0%) |
|  | | | | |

| Table 154 Symptoms questionnaire NOSE |
| --- |

|  | | **Mild intermittent rhinitis N=126** | **Severe intermittent rhinitis N=30** | **Mild persistent rhinitis N=263** | **Severe persistent rhinitis N=983** |
| --- | --- | --- | --- | --- | --- |
| **NOSE score (0-100)** | N | 124 | 29 | 262 | 978 |
|  | Missing | 2 | 1 | 1 | 5 |
|  | Mean ± ET | 29.4 ± 23.6 | 49.8 ± 25.0 | 41.1 ± 21.9 | 62.9 ± 24.1 |
|  | Median | 28 | 55 | 40 | 65 |
|  | Q1 ; Q3 | 10 ; 50 | 40 ; 60 | 25 ; 55 | 45 ; 85 |
|  | Min. ; Max. | 0 ; 95 | 0 ; 100 | 0 ; 100 | 0 ; 100 |
|  | | | | | |

| 0=Patients without symptoms, 100=Patients with the most intense symptoms possible |
| --- |

| Table 155 NOSE score >50 |
| --- |

|  | | **Mild intermittent rhinitis N=126** | **Severe intermittent rhinitis N=30** | **Mild persistent rhinitis N=263** | **Severe persistent rhinitis N=983** |
| --- | --- | --- | --- | --- | --- |
| **NOSE score (class 1)** | N | 124 | 29 | 262 | 978 |
|  | Missing | 2 | 1 | 1 | 5 |
|  | >50 | 21 (16.9%) | 16 (55.2%) | 78 (29.8%) | 644 (65.8%) |
|  | <=50 | 103 (83.1%) | 13 (44.8%) | 184 (70.2%) | 334 (34.2%) |
|  | | | | | |

| Table 158 DYNACHRON |
| --- |

|  | | **Mild intermittent rhinitis N=126** | **Severe intermittent rhinitis N=30** | **Mild persistent rhinitis N=263** | **Severe persistent rhinitis N=983** |
| --- | --- | --- | --- | --- | --- |
| **Partial DYNACHRON score (0-130)** | N | 120 | 29 | 252 | 952 |
|  | Missing | 6 | 1 | 11 | 31 |
|  | Mean ± ET | 21.9 ± 23.6 | 49.0 ± 35.0 | 31.6 ± 25.7 | 57.8 ± 29.3 |
|  | Median | 15 | 49 | 27 | 59 |
|  | Q1 ; Q3 | 3 ; 31 | 22 ; 66 | 11 ; 45 | 35 ; 80 |
|  | Min. ; Max. | 0 ; 96 | 0 ; 113 | 0 ; 118 | 0 ; 130 |
|  | | | | | |

| Score DYNACHRON (0-130) : the higher the score, The greater the embarrassment |
| --- |

| Table 88 NOSE score in classes |
| --- |

|  | | **Total N=1486** |
| --- | --- | --- |
| **NOSE Score (class 1)** | N | 1476 |
|  | Manquant Missing | 10 |
|  | >50 | 804 (54.5%) |
|  | <=50 | 672 (45.5%) |
|  | | |
| **NOSE Score (class 2)** | N | 1476 |
|  | Manquant Missing | 10 |
|  | >75 | 359 (24.3%) |
|  | ]50;75] | 445 (30.1%) |
|  | ]25;50] | 422 (28.6%) |
|  | <=25 | 250 (16.9%) |
|  | | |

**Table II :**

| Table 87 Symptoms questionnaire NOSE |
| --- |

|  | | **Total N=1486** |
| --- | --- | --- |
| **NOSE score (0-100)** | N | 1476 |
|  | Missing | 10 |
|  | Mean ± ET | 55.4 ± 26.7 |
|  | Median | 55 |
|  | Q1 ; Q3 | 35 ; 75 |
|  | Min. ; Max. | 0 ; 100 |
|  | | |

|  | | **from 5 to 11 years N=439** | **12-17 years N=263** | **Adults N=771** |
| --- | --- | --- | --- | --- |
| **NOSE score (0-100)** | N | 436 | 260 | 767 |
|  | Missing | 3 | 3 | 4 |
|  | Mean ± ET | 50.9 ± 25.8 | 56.4 ± 26.0 | 57.7 ± 27.1 |
|  | Median | 50 | 58 | 60 |
|  | Q1 ; Q3 | 30 ; 70 | 40 ; 75 | 40 ; 80 |
|  | Min. ; Max. | 0 ; 100 | 0 ; 100 | 0 ; 100 |
|  | | | | |

| 0=Patients without symptoms, 100=Patients with the most intense symptoms possible |
| --- |

| Table 89 RHINOQOL |
| --- |

|  | | **Total N=1486** |
| --- | --- | --- |
| **RHINOQOL - Frequency (0-100)** | N | 1415 |
|  | Missing | 71 |
|  | Mean ± ET | 61.6 ± 20.8 |
|  | Median | 63 |
|  | Q1 ; Q3 | 44 ; 75 |
|  | Min. ; Max. | 0 ; 100 |
|  | | |
| **RHINOQOL - Embarrassment (0-100)** | N | 1284 |
|  | Missing | 202 |
|  | Mean ± ET | 62.1 ± 22.4 |
|  | Median | 63 |
|  | Q1 ; Q3 | 47 ; 80 |
|  | Min. ; Max. | 0 ; 100 |
|  | | |
| **RHINOQOL - Impact (0-100)** | N | 1447 |
|  | Missing | 39 |
|  | Mean ± ET | 28.1 ± 21.2 |
|  | Median | 25 |
|  | Q1 ; Q3 | 11 ; 44 |
|  | Min. ; Max. | 0 ; 100 |
|  | | |

|  | | **from 5 to 11 years N=439** | **12-17 years N=263** | **Adults N=771** |
| --- | --- | --- | --- | --- |
| **RHINOQOL - Frequency (0-100)** | N | 420 | 248 | 735 |
|  | Missing | 19 | 15 | 36 |
|  | Mean ± ET | 65.8 ± 20.6 | 60.7 ± 19.8 | 59.6 ± 20.9 |
|  | Median | 69 | 63 | 63 |
|  | Q1 ; Q3 | 50 ; 81 | 44 ; 75 | 44 ; 75 |
|  | Min. ; Max. | 13 ; 100 | 19 ; 100 | 0 ; 100 |
|  | | | | |
| **RHINOQOL - Embarrassment (0-100)** | N | 360 | 229 | 684 |
|  | Missing | 79 | 34 | 87 |
|  | Mean ± ET | 69.0 ± 20.8 | 61.3 ± 20.5 | 58.8 ± 22.9 |
|  | Median | 70 | 60 | 60 |
|  | Q1 ; Q3 | 53 ; 87 | 47 ; 77 | 40 ; 77 |
|  | Min. ; Max. | 0 ; 100 | 7 ; 100 | 0 ; 100 |
|  | | | | |
| **RHINOQOL - Impact (0-100)** | N | 425 | 256 | 755 |
|  | Missing | 14 | 7 | 16 |
|  | Mean ± ET | 21.6 ± 19.0 | 26.8 ± 20.4 | 32.1 ± 21.7 |
|  | Median | 17 | 25 | 31 |
|  | Q1 ; Q3 | 6 ; 33 | 11 ; 39 | 14 ; 47 |
|  | Min. ; Max. | 0 ; 89 | 0 ; 100 | 0 ; 100 |
|  | | | | |

| RhinoQOL score Frequency (0-100) : The higher the score, the less frequent the symptoms are |
| --- |
| RhinoQOL score Embarrassment (0-100) : the higher the score, the less discomfort is |
| RhinoQOL score Impact (0-100) : the higher the score, the more pejorative the impact on quality of life |

| Table 91 DYNACHRON |
| --- |

|  | | **Total N=1486** |
| --- | --- | --- |
| **Partial DYNACHRON score (0-130)** | N | 1432 |
|  | Missing | 54 |
|  | Mean ± ET | 49.4 ± 31.3 |
|  | Median | 47 |
|  | Q1 ; Q3 | 25 ; 74 |
|  | Min. ; Max. | 0 ; 130 |
|  | | |

|  | | **from 5 to 11 years N=439** | **12-17 years N=263** | **Adults N=771** |
| --- | --- | --- | --- | --- |
| **Partial DYNACHRON score (0-130)** | N | 424 | 251 | 745 |
|  | Missing | 15 | 12 | 26 |
|  | Mean ± ET | 41.9 ± 28.3 | 51.9 ± 30.8 | 52.9 ± 32.4 |
|  | Median | 38 | 51 | 54 |
|  | Q1 ; Q3 | 19 ; 63 | 27 ; 76 | 27 ; 77 |
|  | Min. ; Max. | 0 ; 120 | 0 ; 130 | 0 ; 130 |
|  | | | | |

| DYNACHRON score (0-130) : the higher the score, the greater the embarrassment |
| --- |

| Table 92 If asthmatic patient (Adult or child 12 years of age or older), ACQ score |
| --- |

|  | | **Total N=303** |
| --- | --- | --- |
| **ACQ score** | N | 285 |
|  | Missing | 18 |
|  | Mean ± ET | 1.4 ± 1.1 |
|  | Median | 1 |
|  | Q1 ; Q3 | 1 ; 2 |
|  | Min. ; Max. | 0 ; 6 |
|  | | |

|  | | **12-17 years N=91** | **Adults N=212** |
| --- | --- | --- | --- |
| **ACQ score** | N | 84 | 201 |
|  | Missing | 7 | 11 |
|  | Mean ± ET | 1.2 ± 1.0 | 1.5 ± 1.2 |
|  | Median | 1 | 1 |
|  | Q1 ; Q3 | 0 ; 2 | 1 ; 2 |
|  | Min. ; Max. | 0 ; 4 | 0 ; 6 |
|  | | | |

| Means of the 6 items (from 0-6, score 6 is the worst) |
| --- |

| Table 95* Number of exacerbations in the past 12 months ** Outliers of exacerbations (>50) were considered "missing".* | | | |
| --- | --- | --- | --- |
|  | | **Total N=1486** |  |
| **How many times in the past 12 months** | N | 1166 |  |
|  | Missing | 320 |  |
|  | 0 | 260 (22.3%) |  |
|  | 1 | 270 (23.2%) |  |
|  | 2 | 212 (18.2%) |  |
|  | 3 | 184 (15.8%) |  |
|  | 4 | 67 (5.7%) |  |
|  | 5 | 89 (7.6%) |  |
|  | 6 | 14 (1.2%) |  |
|  | 7 | 7 (0.6%) |  |
|  | 8 | 8 (0.7%) |  |
|  | 10 | 17 (1.5%) |  |
|  | 12 | 24 (2.1%) |  |
|  | 14 | 1 (0.1%) |  |
|  | 20 | 3 (0.3%) |  |
|  | 24 | 4 (0.3%) |  |
|  | 25 | 1 (0.1%) |  |
|  | 27 | 1 (0.1%) |  |
|  | 30 | 1 (0.1%) |  |
|  | 40 | 1 (0.1%) |  |
|  | 50 | 2 (0.2%) |  |
|  | | |  |

|  | | **from 5 to 11 years N=439** | **12-17 years N=263** | **Adults N=771** |
| --- | --- | --- | --- | --- |
| **How many times in the past 12 months** | N | 337 | 196 | 623 |
|  | Missing | 102 | 67 | 148 |
|  | 0 | 87 (25.8%) | 47 (24.0%) | 125 (20.1%) |
|  | 1 | 59 (17.5%) | 49 (25.0%) | 160 (25.7%) |
|  | 2 | 67 (19.9%) | 33 (16.8%) | 110 (17.7%) |
|  | 3 | 66 (19.6%) | 29 (14.8%) | 88 (14.1%) |
|  | 4 | 12 (3.6%) | 16 (8.2%) | 37 (6.0%) |
|  | 5 | 24 (7.1%) | 14 (7.1%) | 50 (8.0%) |
|  | 6 | 4 (1.2%) | 1 (0.5%) | 9 (1.4%) |
|  | 7 | 4 (1.2%) | 0 (0.0%) | 3 (0.5%) |
|  | 8 | 3 (0.9%) | 1 (0.5%) | 4 (0.6%) |
|  | 10 | 4 (1.2%) | 1 (0.5%) | 12 (1.9%) |
|  | 12 | 5 (1.5%) | 3 (1.5%) | 16 (2.6%) |
|  | 14 | 1 (0.3%) | 0 (0.0%) | 0 (0.0%) |
|  | 20 | 0 (0.0%) | 0 (0.0%) | 2 (0.3%) |
|  | 24 | 0 (0.0%) | 2 (1.0%) | 2 (0.3%) |
|  | 25 | 0 (0.0%) | 0 (0.0%) | 1 (0.2%) |
|  | 27 | 0 (0.0%) | 0 (0.0%) | 1 (0.2%) |
|  | 30 | 0 (0.0%) | 0 (0.0%) | 1 (0.2%) |
|  | 40 | 0 (0.0%) | 0 (0.0%) | 1 (0.2%) |
|  | 50 | 1 (0.3%) | 0 (0.0%) | 1 (0.2%) |
|  | | | | |

#### Table 102b* Impact on quality of life - In patients with at least 2 exacerbations

|  | **Total (N=636)** | | |  |  |  |  |  |  |  |
| --- | --- | --- | --- | --- | --- | --- | --- | --- | --- | --- |
|  | **n (1)** | **% (2)** | |  |  |  |  |  |  |  |
| TOTAL | 598 | 94.0 | | |  |  |  |  |  |  |
| MODERATELY DEGRADED | 411 | 64.6 | | |  |  |  |  |  |  |
| VERY DEGRADED | 251 | 39.5 | | |  |  |  |  |  |  |
| UNCHANGED | 98 | 15.4 | | |  |  |  |  |  |  |
| IMPROVED | 25 | 3.9 | | |  |  |  |  |  |  |
|  | | | | | | | | | | |
| (1) Number of patients with at least impact | | | | | | | | | | |
| (2) (n/N)*100 (N: Number of patients) | | | | | | | | | | |
|  | | | | | | | | | | |
|  | **from 5 to 11 years (N=191)** | | | | | **12-17 years (N=100)** | | **Adults (N=338)** | |  |
|  | **n (1)** | | **% (2)** | | | **n (1)** | **n (1)** | **% (2)** | **n (1)** |  |
| TOTAL | 178 | | 93.19 | | | 91 | 91.00 | 322 | 95.27 |  |
| MODERATELY DEGRADED | 114 | | 59.69 | | | 65 | 65.00 | 225 | 66.57 |  |
| VERY DEGRADED | 65 | | 34.03 | | | 32 | 32.00 | 152 | 44.97 |  |
| UNCHANGED | 42 | | 21.99 | | | 19 | 19.00 | 37 | 10.95 |  |
| IMPROVED | 10 | | 5.24 | | | 3 | 3.00 | 11 | 3.25 |  |
|  | | | | | | | | | | |
| (1) Number of patients with at least impact | | | | | | | | | | |
| (2) (n/N)*100 (N: Number of patients per group) | | | | | | | | | | |

**Table III :**

| Table 96* Number of exacerbations in the past 12 months (ongoing) |
| --- |

** Outliers of exacerbations (>50) were considered "missing".*

|  | | **Total N=1486** |
| --- | --- | --- |
| **How many times in the past 12 months** | N | 1166 |
|  | Missing | 320 |
|  | Mean ± ET | 2.6 ± 3.9 |
|  | Median | 2 |
|  | Min. ; Max. | 0 ; 50 |
|  | | |

|  | | **from 5 to 11 years N=439** | **12-17 years N=263** | **Adults N=771** |
| --- | --- | --- | --- | --- |
| **How many times in the past 12 months** | N | 337 | 196 | 623 |
|  | Missing | 102 | 67 | 148 |
|  | Mean ± ET | 2.4 ± 3.5 | 2.3 ± 3.1 | 2.8 ± 4.2 |
|  | Median | 2 | 2 | 2 |
|  | Min. ; Max. | 0 ; 50 | 0 ; 24 | 0 ; 50 |
|  | | | | |

| Table 96* Did you take additional treatment ? |
| --- |

|  | | **Total N=1486** |
| --- | --- | --- |
| **Did you take additional treatment ?** | N | 1228 |
|  | Missing | 258 |
|  | No | 408 (33.2%) |
|  | Yes | 820 (66.8%) |
|  | | |

|  | | **from 5 to 11 years N=439** | **From 12 to 17 years N=263** | **Adults N=771** |
| --- | --- | --- | --- | --- |
| **Did you take additional treatment ?** | N | 364 | 206 | 645 |
|  | Missing | 75 | 57 | 126 |
|  | No | 118 (32.4%) | 78 (37.9%) | 207 (32.1%) |
|  | Yes | 246 (67.6%) | 128 (62.1%) | 438 (67.9%) |
|  | | | | |

| Table 183* Predictors of AR exacerbations - At least 2 exacerbations |
| --- |

** Outliers of exacerbations (>50) were considered "missing".*

|  | | **Total N=1166** | **No N=530** | **Yes N=636** | **P-value Univariate** | **OR univarié** | **P-value Multivariate**** | **OR multivariate** |
| --- | --- | --- | --- | --- | --- | --- | --- | --- |
| **Sex** | Missing | 5 | 4 | 1 |  |  |  |  |
|  | M | 599 (100.0%) | 283 (47.2%) | 316 (52.8%) | **0.171** * | Ref |  |  |
|  | F | 562 (100.0%) | 243 (43.2%) | 319 (56.8%) |  | 1.18 [0.93 ; 1.48] |  |  |
| **Age** | Missing | 10 | 3 | 7 |  |  |  |  |
|  | from 5 to 11 years | 337 (100.0%) | 146 (43.3%) | 191 (56.7%) | 0.447 | Ref |  |  |
|  | 12-17 years | 196 (100.0%) | 96 (49.0%) | 100 (51.0%) |  | 0.80 [0.56 ; 1.13] |  |  |
|  | Adults | 623 (100.0%) | 285 (45.8%) | 338 (54.2%) |  | 0.91 [0.69 ; 1.18] |  |  |
| **Smoking** | Missing | 7 | 3 | 4 |  |  |  |  |
|  | No | 1082 (100.0%) | 491 (45.4%) | 591 (54.6%) | 0.815 | Ref |  |  |
|  | Yes | 77 (100.0%) | 36 (46.8%) | 41 (53.2%) |  | 0.95 [0.60 ; 1.50] |  |  |
| **Use of cannabis** | Missing | 10 | 3 | 7 |  |  |  |  |
|  | No | 1147 (100.0%) | 521 (45.4%) | 626 (54.6%) | 0.217 | Ref |  |  |
|  | Yes | 9 (100.0%) | 6 (66.7%) | 3 (33.3%) |  | 0.42 [0.10 ; 1.67] |  |  |
| **Conjunctivitis** | No | 515 (100.0%) | 254 (49.3%) | 261 (50.7%) | **0.019** * | Ref |  |  |
|  | Yes | 651 (100.0%) | 276 (42.4%) | 375 (57.6%) |  | 1.32 [1.05 ; 1.67] |  |  |
| **Sinusitis** | No | 760 (100.0%) | 364 (47.9%) | 396 (52.1%) | **0.022** * | Ref |  |  |
|  | Yes | 406 (100.0%) | 166 (40.9%) | 240 (59.1%) |  | 1.33 [1.04 ; 1.70] |  |  |
| **Atopic eczema** | No | 931 (100.0%) | 434 (46.6%) | 497 (53.4%) | **0.113** * | Ref |  |  |
|  | Yes | 235 (100.0%) | 96 (40.9%) | 139 (59.1%) |  | 1.26 [0.95 ; 1.69] |  |  |
| **Food allergy** | No | 1062 (100.0%) | 487 (45.9%) | 575 (54.1%) | 0.379 | Ref |  |  |
|  | Yes | 104 (100.0%) | 43 (41.3%) | 61 (58.7%) |  | 1.20 [0.80 ; 1.81] |  |  |
| **Allergic urticaria** | No | 1083 (100.0%) | 493 (45.5%) | 590 (54.5%) | 0.868 | Ref |  |  |
|  | Yes | 83 (100.0%) | 37 (44.6%) | 46 (55.4%) |  | 1.04 [0.66 ; 1.63] |  |  |
| **Age of diagnosis de la RA** | Missing | 12 | 7 | 5 |  |  |  |  |
|  | <=1 years | 700 (100.0%) | 335 (47.9%) | 365 (52.1%) | **0.032** * | Ref |  |  |
|  | >1 years | 454 (100.0%) | 188 (41.4%) | 266 (58.6%) |  | 1.30 [1.02 ; 1.65] |  |  |
| **Polysensibilisation Polysensitization (Y/N)** | Missing | 14 | 6 | 8 |  |  |  |  |
|  | No | 533 (100.0%) | 274 (51.4%) | 259 (48.6%) | **<0.001** * | Ref | **0.001** | Ref |
|  | Yes | 619 (100.0%) | 250 (40.4%) | 369 (59.6%) |  | 1.56 [1.24 ; 1.97] |  | 1.57 [1.19 ; 2.07] |
| **Food allergy** | Missing | 16 | 9 | 7 |  |  |  |  |
|  | No | 1061 (100.0%) | 486 (45.8%) | 575 (54.2%) | 0.239 | Ref |  |  |
|  | Yes | 89 (100.0%) | 35 (39.3%) | 54 (60.7%) |  | 1.30 [0.84 ; 2.03] |  |  |

####

#### Table 99b* Average duration of symptoms (jours) - In patients with at least 2 exacerbations

|  | | **Total N=636** |
| --- | --- | --- |
| **Average duration of symptoms (jours)** | N | 592 |
|  | Missing | 44 |
|  | Mean ± ET | 14.5 ± 16.7 |
|  | Median | 10.00 |
|  | Min. ; Max. | 1 ; 185 |
|  | | |

|  | | **from 5 to 11 years N=191** | **12-17 years N=100** | **Adults N=338** |
| --- | --- | --- | --- | --- |
| **Average duration of symptoms (jours)** | N | 180 | 87 | 319 |
|  | Missing | 11 | 13 | 38 |
|  | Mean ± ET | 12.7 ± 12.3 | 13.6 ± 15.2 | 15.6 ± 19.1 |
|  | Median | 8.67 | 8.40 | 10.20 |
|  | Min. ; Max. | 1 ; 90 | 1 ; 105 | 1 ; 185 |
|  | | | | |

#### Table 100b* Main symptoms - In patients with at least 2 exacerbations

|  | **Total (N=636)** | |  |
| --- | --- | --- | --- |
|  | **n (1)** | **% (2)** |  |
| TOTAL | 631 | 99.2 |  |
| SNEEZING | 545 | 85.7 |  |
| RUNNY NOSE | 541 | 85.1 |  |
| STUFFY NOSE | 540 | 84.9 |  |
| CONJONCTIVITIS | 267 | 42.0 |  |
| LOSS OF SMELL | 195 | 30.7 |  |
|  | | | |
| (1) Number of patients with at least one symptom | | | |
| (2) (n/N)*100 (N: Number of patients) | | | |
|  | | | |

|  | **from 5 to 11 years (N=191)** | | **12-17 years (N=100)** | | **Adults (N=338)** | |  |
| --- | --- | --- | --- | --- | --- | --- | --- |
|  | **n (1)** | **% (2)** | **n (1)** | **n (1)** | **% (2)** | **n (1)** |  |
| TOTAL | 189 | 98.95 | 100 | 100.00 | 335 | 99.11 |  |
| SNEEZING | 161 | 84.29 | 91 | 91.00 | 287 | 84.91 |  |
| RUNNY NOSE | 156 | 81.68 | 90 | 90.00 | 288 | 85.21 |  |
| STUFFY NOSE | 162 | 84.82 | 90 | 90.00 | 281 | 83.14 |  |
| CONJONCTIVITIS | 75 | 39.27 | 46 | 46.00 | 145 | 42.90 |  |
| LOSS OF SMELL | 32 | 16.75 | 30 | 30.00 | 131 | 38.76 |  |
|  | | | | | | | |
| (1) Number of patients with at least one symptom | | | | | | | |
| (2) (n/N)*100 (N: Number of patients per group) | | | | | | | |

|  | **n (1)** | **% (2)** | |  |  |  |  |  |  |
| --- | --- | --- | --- | --- | --- | --- | --- | --- | --- |
| TOTAL | 515 | 56.8 | |  |  |  |  |  |  |
| INFECTION | 303 | 33.4 | |  |  |  |  |  |  |
| POLLUTION | 227 | 25.1 | |  |  |  |  |  |  |
| STRESS | 151 | 16.7 | |  |  |  |  |  |  |
| TOBACCO | 68 | 7.5 | |  |  |  |  |  |  |
| OCCUPATIONAL EXPOSURE | 43 | 4.7 | |  |  |  |  |  |  |
|  | | | | | | | | | |
| (1) Number of patients with at least one factor | | | | | | | | | |
| (2) (n/N)*100 (N: Number of patients) | | | | | | | | | |
|  | | | | | | | | | |
|  | **from 5 to 11 years (N=250)** | | | | **12-17 years (N=149)** | | **Adults (N=498)** | |  |
|  | **n (1)** | | **% (2)** | | **n (1)** | **n (1)** | **% (2)** | **n (1)** |  |
| TOTAL | 126 | | 50.40 | | 82 | 55.03 | 301 | 60.44 |  |
| INFECTION | 99 | | 39.60 | | 44 | 29.53 | 158 | 31.73 |  |
| POLLUTION | 48 | | 19.20 | | 39 | 26.17 | 137 | 27.51 |  |
| STRESS | 15 | | 6.00 | | 19 | 12.75 | 116 | 23.29 |  |
| TOBACCO | 7 | | 2.80 | | 13 | 8.72 | 47 | 9.44 |  |
| OCCUPATIONAL EXPOSURE | 1 | | 0.40 | | 4 | 2.68 | 38 | 7.63 |  |
|  | | | | | | | | | |
| (1) Number of patients with at least one factor | | | | | | | | | |
| (2) (n/N)*100 (N: Number of patients per group) | | | | | | | | | |
|  | | | | | | | | | |

#### Table 101b* Aggravating factors - In patients with at least 2 exacerbations

|  | **Total (N=636)** | |  |
| --- | --- | --- | --- |
|  | **n (1)** | **% (2)** |  |
| TOTAL | 384 | 60.4 |  |
| INFECTION | 241 | 37.9 |  |
| POLLUTION | 176 | 27.7 |  |
| STRESS | 126 | 19.8 |  |
| TOBACCO | 47 | 7.4 |  |
| OCCUPATIONAL EXPOSURE | 34 | 5.3 |  |
|  | | | |
| (1) Number of patients with at least one factor | | | |
| (2) (n/N)*100 (N: Number of patients) | | | |
|  | | | |

|  | **from 5 to 11 years (N=191)** | | **12-17 years (N=100)** | | **Adults (N=338)** | |  |
| --- | --- | --- | --- | --- | --- | --- | --- |
|  | **n (1)** | **% (2)** | **n (1)** | **n (1)** | **% (2)** | **n (1)** |  |
| TOTAL | 100 | 52.36 | 58 | 58.00 | 220 | 65.09 |  |
| INFECTION | 79 | 41.36 | 33 | 33.00 | 127 | 37.57 |  |
| POLLUTION | 39 | 20.42 | 28 | 28.00 | 106 | 31.36 |  |
| STRESS | 14 | 7.33 | 15 | 15.00 | 96 | 28.40 |  |
| TOBACCO | 4 | 2.09 | 8 | 8.00 | 34 | 10.06 |  |
| OCCUPATIONAL EXPOSURE | 1 | 0.52 | 3 | 3.00 | 30 | 8.88 |  |
|  | | | | | | | |
| (1) Number of patients with at least one factor | | | | | | | |
| (2) (n/N)*100 (N: Number of patients per group) | | | | | | | |

####

#### Table 100b* Main symptoms- In patients with at least 2 exacerbations

|  | **Total (N=636)** | |  |
| --- | --- | --- | --- |
|  | **n (1)** | **% (2)** |  |
| TOTAL | 631 | 99.2 |  |
| SNEEZING | 545 | 85.7 |  |
| RUNNY NOSE | 541 | 85.1 |  |
| STUFFY NOSE | 540 | 84.9 |  |
| CONJONCTIVITIS | 267 | 42.0 |  |
| LOSS OF SMELL | 195 | 30.7 |  |
|  | | | |
| (1) Number of patients with at least one symptom | | | |
| (2) (n/N)*100 (N: Number of patients) | | | |
|  | | | |

|  | **from 5 to 11 years (N=191)** | | **12-17 years (N=100)** | | **Adults (N=338)** | |  |
| --- | --- | --- | --- | --- | --- | --- | --- |
|  | **n (1)** | **% (2)** | **n (1)** | **n (1)** | **% (2)** | **n (1)** |  |
| TOTAL | 189 | 98.95 | 100 | 100.00 | 335 | 99.11 |  |
| SNEEZING | 161 | 84.29 | 91 | 91.00 | 287 | 84.91 |  |
| RUNNY NOSE | 156 | 81.68 | 90 | 90.00 | 288 | 85.21 |  |
| STUFFY NOSE | 162 | 84.82 | 90 | 90.00 | 281 | 83.14 |  |
| CONJONCTIVITIS | 75 | 39.27 | 46 | 46.00 | 145 | 42.90 |  |
| LOSS OF SMELL | 32 | 16.75 | 30 | 30.00 | 131 | 38.76 |  |
|  | | | | | | | |
| (1) Number of patients with at least one factor | | | | | | | |
| (2) (n/N)*100 (N: Number of patients per group) | | | | | | | |

####

#### Table 102b* Impact on quality of life - In patients with at least 2 exacerbations

|  | **Total (N=636)** | | |  |  |  |  |  |  |  |
| --- | --- | --- | --- | --- | --- | --- | --- | --- | --- | --- |
|  | **n (1)** | **% (2)** | |  |  |  |  |  |  |  |
| TOTAL | 598 | 94.0 | | |  |  |  |  |  |  |
| MODERATELY DEGRADED | 411 | 64.6 | | |  |  |  |  |  |  |
| VERY DEGRADED | 251 | 39.5 | | |  |  |  |  |  |  |
| UNCHANGED | 98 | 15.4 | | |  |  |  |  |  |  |
| IMPROVED | 25 | 3.9 | | |  |  |  |  |  |  |
|  | | | | | | | | | | |
| (1) Number of patients with at least impact | | | | | | | | | | |
| (2) (n/N)*100 (N: Number of patients) | | | | | | | | | | |
|  | | | | | | | | | | |
|  | **from 5 to 11 years (N=191)** | | | | | **12-17 years (N=100)** | | **Adults (N=338)** | |  |
|  | **n (1)** | | **% (2)** | | | **n (1)** | **n (1)** | **% (2)** | **n (1)** |  |
| TOTAL | 178 | | 93.19 | | | 91 | 91.00 | 322 | 95.27 |  |
| MODERATELY DEGRADED | 114 | | 59.69 | | | 65 | 65.00 | 225 | 66.57 |  |
| VERY DEGRADED | 65 | | 34.03 | | | 32 | 32.00 | 152 | 44.97 |  |
| UNCHANGED | 42 | | 21.99 | | | 19 | 19.00 | 37 | 10.95 |  |
| IMPROVED | 10 | | 5.24 | | | 3 | 3.00 | 11 | 3.25 |  |
|  | | | | | | | | | | |
| (1) Number of patients with at least impact | | | | | | | | | | |
| (2) (n/N)*100 (N: Number of patients per group) | | | | | | | | | | |

**Table IV:**

| Table 183* Predictors of AR exacerbations - At least 2 exacerbations |
| --- |

** Outliers of exacerbations (>50) were considered "missing".*

|  | | **Total N=1166** | **No N=530** | **Yes N=636** | **P-value Univariate** | **OR univariate** | **P-value Multivariate**** | **OR multivariate** |
| --- | --- | --- | --- | --- | --- | --- | --- | --- |
| **Sex** | Missing | 5 | 4 | 1 |  |  |  |  |
|  | M | 599 (100.0%) | 283 (47.2%) | 316 (52.8%) | **0.171** * | Ref |  |  |
|  | F | 562 (100.0%) | 243 (43.2%) | 319 (56.8%) |  | 1.18 [0.93 ; 1.48] |  |  |
| **Age** | Missing | 10 | 3 | 7 |  |  |  |  |
|  | from 5 to 11 years | 337 (100.0%) | 146 (43.3%) | 191 (56.7%) | 0.447 | Ref |  |  |
|  | 12-17 years | 196 (100.0%) | 96 (49.0%) | 100 (51.0%) |  | 0.80 [0.56 ; 1.13] |  |  |
|  | Adults | 623 (100.0%) | 285 (45.8%) | 338 (54.2%) |  | 0.91 [0.69 ; 1.18] |  |  |
| **Smoking** | Missing | 7 | 3 | 4 |  |  |  |  |
|  | No | 1082 (100.0%) | 491 (45.4%) | 591 (54.6%) | 0.815 | Ref |  |  |
|  | Yes | 77 (100.0%) | 36 (46.8%) | 41 (53.2%) |  | 0.95 [0.60 ; 1.50] |  |  |
| **Use of cannabis** | Missing | 10 | 3 | 7 |  |  |  |  |
|  | No | 1147 (100.0%) | 521 (45.4%) | 626 (54.6%) | 0.217 | Ref |  |  |
|  | Yes | 9 (100.0%) | 6 (66.7%) | 3 (33.3%) |  | 0.42 [0.10 ; 1.67] |  |  |
| **Conjunctivitis** | No | 515 (100.0%) | 254 (49.3%) | 261 (50.7%) | **0.019** * | Ref |  |  |
|  | Yes | 651 (100.0%) | 276 (42.4%) | 375 (57.6%) |  | 1.32 [1.05 ; 1.67] |  |  |
| **Sinusitis** | No | 760 (100.0%) | 364 (47.9%) | 396 (52.1%) | **0.022** * | Ref |  |  |
|  | Yes | 406 (100.0%) | 166 (40.9%) | 240 (59.1%) |  | 1.33 [1.04 ; 1.70] |  |  |
| **Atopic eczema** | No | 931 (100.0%) | 434 (46.6%) | 497 (53.4%) | **0.113** * | Ref |  |  |
|  | Yes | 235 (100.0%) | 96 (40.9%) | 139 (59.1%) |  | 1.26 [0.95 ; 1.69] |  |  |
| **Food allergy** | No | 1062 (100.0%) | 487 (45.9%) | 575 (54.1%) | 0.379 | Ref |  |  |
|  | Yes | 104 (100.0%) | 43 (41.3%) | 61 (58.7%) |  | 1.20 [0.80 ; 1.81] |  |  |
| **Allergic urticaria** | No | 1083 (100.0%) | 493 (45.5%) | 590 (54.5%) | 0.868 | Ref |  |  |
|  | Yes | 83 (100.0%) | 37 (44.6%) | 46 (55.4%) |  | 1.04 [0.66 ; 1.63] |  |  |
| **Age of AR diagnosis diagnosis de la RA** | Missing | 12 | 7 | 5 |  |  |  |  |
|  | <=1 years | 700 (100.0%) | 335 (47.9%) | 365 (52.1%) | **0.032** * | Ref |  |  |
|  | >1 years | 454 (100.0%) | 188 (41.4%) | 266 (58.6%) |  | 1.30 [1.02 ; 1.65] |  |  |
| **Polysensitisation (Y/N)** | Missing | 14 | 6 | 8 |  |  |  |  |
|  | No | 533 (100.0%) | 274 (51.4%) | 259 (48.6%) | **<0.001** * | Ref | **0.001** | Ref |
|  | Yes | 619 (100.0%) | 250 (40.4%) | 369 (59.6%) |  | 1.56 [1.24 ; 1.97] |  | 1.57 [1.19 ; 2.07] |
| **Food allergy** | Missing | 16 | 9 | 7 |  |  |  |  |
|  | No | 1061 (100.0%) | 486 (45.8%) | 575 (54.2%) | 0.239 | Ref |  |  |
|  | Yes | 89 (100.0%) | 35 (39.3%) | 54 (60.7%) |  | 1.30 [0.84 ; 2.03] |  |  |
| **Allergy to Hymenoptera venoms** | Missing | 192 | 105 | 87 |  |  |  |  |
|  | No | 971 (100.0%) | 424 (43.7%) | 547 (56.3%) | 0.721 | Ref |  |  |
|  | Yes | 3 (100.0%) | 1 (33.3%) | 2 (66.7%) |  | 1.55 [0.14 ; 17.15] |  |  |
| **Drug allergy** | Missing | 197 | 106 | 91 |  |  |  |  |
|  | No | 944 (100.0%) | 414 (43.9%) | 530 (56.1%) | 0.702 | Ref |  |  |
|  | Yes | 25 (100.0%) | 10 (40.0%) | 15 (60.0%) |  | 1.17 [0.52 ; 2.63] |  |  |
| **Occupational allergy** | Missing | 196 | 106 | 90 |  |  |  |  |
|  | No | 964 (100.0%) | 420 (43.6%) | 544 (56.4%) | 0.273 | Ref |  |  |
|  | Yes | 6 (100.0%) | 4 (66.7%) | 2 (33.3%) |  | 0.39 [0.07 ; 2.12] |  |  |
| **ARIA classification** | Missing | 65 | 25 | 40 |  |  |  |  |
|  | Mild persistent rhinitis | 200 (100.0%) | 125 (62.5%) | 75 (37.5%) | **<0.001** * | Ref | **0.002** | Ref |
|  | Mild intermittent rhinitis | 100 (100.0%) | 53 (53.0%) | 47 (47.0%) |  | 1.48 [0.91 ; 2.40] |  | 1.77 [1.02 ; 3.10] |
|  | Severe persistent rhinitis | 777 (100.0%) | 320 (41.1%) | 457 (58.2%) |  | 2.38 [1.73 ; 3.28] |  | 1.91 [1.30 ; 2.80] |
|  | Severe intermittent rhinitis | 24 (100.0%) | 7 (29.2%) | 17 (70.8%) |  | 4.05 [1.60 ; 10.21] |  | 5.34 [1.66 ; 17.11] |
| **Sneezing** | No | 43 (100.0%) | 21 (48.8%) | 22 (51.2%) | 0.649 | Ref |  |  |
|  | Yes | 1123 (100.0%) | 509 (45.3%) | 614 (54.7%) |  | 1.15 [0.63 ; 2.12] |  |  |
| **Rhinorrhoea** | No | 38 (100.0%) | 20 (52.6%) | 18 (47.4%) | 0.368 | Ref |  |  |
|  | Yes | 1128 (100.0%) | 510 (45.2%) | 618 (54.8%) |  | 1.35 [0.70 ; 2.57] |  |  |
| **Nasal obstruction** | No | 74 (100.0%) | 35 (47.3%) | 41 (52.7%) | 0.742 | Ref |  |  |
|  | Yes | 1092 (100.0%) | 495 (45.3%) | 597 (54.7%) |  | 1.08 [0.68 ; 1.74] |  |  |
| **Nasal pruritus** | No | 216 (100.0%) | 110 (50.9%) | 106 (49.1%) | **0.074** * | Ref |  |  |
|  | Yes | 958 (100.0%) | 420 (43.8%) | 538 (56.2%) |  | 1.31 [0.97 ; 1.76] |  |  |
| **Loss of smell** | No | 664 (100.0%) | 326 (49.1%) | 338 (53.1%) | **0.004** * | Ref |  |  |
|  | Yes | 502 (100.0%) | 204 (40.6%) | 298 (59.4%) |  | 1.41 [1.11 ; 1.78] |  |  |
| **Ocular pruritus** | No | 471 (100.0%) | 224 (47.6%) | 247 (52.4%) | 0.235 | Ref |  |  |
|  | Yes | 695 (100.0%) | 306 (44.0%) | 389 (56.0%) |  | 1.15 [0.91 ; 1.46] |  |  |
| **Redness and tearing** | No | 567 (100.0%) | 277 (48.9%) | 290 (51.1%) | **0.023** * | Ref |  |  |
|  | Yes | 599 (100.0%) | 253 (42.2%) | 352 (57.8%) |  | 1.31 [1.04 ; 1.65] |  |  |
| **Asthmatic patient (Y/N)** | Missing | 25 | 9 | 16 |  |  |  |  |
|  | No | 752 (100.0%) | 354 (47.1%) | 398 (52.9%) | **0.183** * | Ref |  |  |
|  | Yes | 389 (100.0%) | 167 (42.9%) | 222 (57.1%) |  | 1.18 [0.92 ; 1.51] |  |  |
| **NOSE score (class 1)** | Missing | 7 | 2 | 5 |  |  |  |  |
|  | <=50 | 523 (100.0%) | 295 (56.4%) | 228 (43.6%) | **<0.001** * | Ref | **<0.001** | Ref |
|  | > 50 | 636 (100.0%) | 233 (36.6%) | 403 (63.4%) |  | 2.24 [1.77 ; 2.84] |  | 1.92 [1.43 ; 2.57] |
| **RHINOQOL - Frequency** | Missing | 47 | 23 | 24 |  |  |  |  |
|  | >50 | 717 (100.0%) | 359 (50.1%) | 358 (49.9%) | **<0.001** * | Ref |  |  |
|  | <=50 | 402 (100.0%) | 148 (36.8%) | 254 (63.2%) |  | 1.72 [1.34 ; 2.21] |  |  |
| **RHINOQOL - Embarrassment** | Missing | 145 | 77 | 69 |  |  |  |  |
|  | >50 | 670 (100.0%) | 335 (50.0%) | 335 (50.0%) | **<0.001** * | Ref |  |  |
|  | <=50 | 351 (100.0%) | 118 (33.6%) | 237 (66.4%) |  | 1.97 [1.51 ; 2.58] |  |  |
| **RHINOQOL - Impact** | Missing | 25 | 7 | 18 |  |  |  |  |
|  | <=50 | 956 (100.0%) | 462 (48.3%) | 494 (51.7%) | **<0.001** * | Ref |  |  |
|  | > 50 | 965 (100.0%) | 61 (33.0%) | 124 (67.0%) |  | 1.90 [1.36 ; 2.64] |  |  |
| ** All variables with a p-value univariate <= 0.20 and with less than 20% missing data were selected for multivariate analysis ** "Stepwise" method with entry threshold at 0.20% and exit threshold at 0.05%* | | | | | | | | |

| Table 134 If asthmatic patient, Mini AQLQ |
| --- |

|  | | **Well controlled N=238** | **Partially controlled N=106** | **Poorly controlled N=65** |
| --- | --- | --- | --- | --- |
| **Mini AQLQ – Symptoms (1-7)** | N | 203 | 96 | 60 |
|  | Missing | 35 | 10 | 5 |
|  | Mean ± ET | 5.2 ± 1.1 | 4.6 ± 1.2 | 3.9 ± 1.2 |
|  | Median | 5 | 5 | 4 |
|  | Q1 ; Q3 | 4 ; 6 | 4 ; 5 | 3 ; 5 |
|  | Min. ; Max. | 1 ; 7 | 2 ; 7 | 1 ; 7 |
|  | | | | |
| **Mini AQLQ – Environment (1-7)** | N | 199 | 94 | 59 |
|  | Missing | 39 | 12 | 6 |
|  | Mean ± ET | 4.9 ± 1.4 | 4.5 ± 1.4 | 4.3 ± 1.3 |
|  | Median | 5 | 5 | 4 |
|  | Q1 ; Q3 | 4 ; 6 | 3 ; 6 | 3 ; 5 |
|  | Min. ; Max. | 1 ; 7 | 1 ; 7 | 2 ; 7 |
|  | | | | |
| **Mini AQLQ - Emotion (1-7)** | N | 203 | 96 | 60 |
|  | Missing | 35 | 10 | 5 |
|  | Mean ± ET | 5.4 ± 1.4 | 4.9 ± 1.4 | 4.5 ± 1.4 |
|  | Median | 6 | 5 | 4 |
|  | Q1 ; Q3 | 4 ; 7 | 4 ; 6 | 3 ; 5 |
|  | Min. ; Max. | 1 ; 7 | 2 ; 7 | 2 ; 7 |
|  | | | | |
| **Mini AQLQ - Activities (1-7)** | N | 203 | 96 | 61 |
|  | Missing | 35 | 10 | 4 |
|  | Mean ± ET | 5.6 ± 1.3 | 5.2 ± 1.3 | 4.6 ± 1.5 |
|  | Median | 6 | 5 | 5 |
|  | Q1 ; Q3 | 5 ; 7 | 4 ; 6 | 4 ; 6 |
|  | Min. ; Max. | 1 ; 7 | 2 ; 7 | 1 ; 7 |
|  | | | | |
| **Mini AQLQ - Total score(1-7)** | N | 187 | 88 | 57 |
|  | Missing | 51 | 18 | 8 |
|  | Mean ± ET | 5.3 ± 1.1 | 4.8 ± 1.1 | 4.3 ± 1.2 |
|  | Median | 5 | 5 | 4 |
|  | Q1 ; Q3 | 5 ; 6 | 4 ; 6 | 4 ; 5 |
|  | Min. ; Max. | 1 ; 7 | 3 ; 7 | 2 ; 7 |
|  | | | | |

| Means of domain items: score 1 is the worst |
| --- |

| Table 185* Predictors of asthma exacerbations- At least 2 exacerbations |
| --- |

** aberrant numbers of severe No exacerbations (>50) were considered "missing".*

|  | | **Total N=443** | **No N=249** | **Yes N=194** | **P-value Univariate** | **OR univariate** | **P-value Multivariate**** | **OR multivariate** |
| --- | --- | --- | --- | --- | --- | --- | --- | --- |
| **Sex** | Missing | 1 | 1 | 0 |  |  |  |  |
|  | M | 235 (100.0%) | 134 (57.0%) | 101 (43.0%) | 0.680 | Ref |  |  |
|  | F | 207 (100.0%) | 114 (55.1%) | 93 (44.9%) |  | 1.08 [0.74 ; 1.58] |  |  |
| **Age** | Missing | 3 | 2 | 1 |  |  |  |  |
|  | from 5 to 11 years | 170 (100.0%) | 92 (54.1%) | 78 (45.9%) | 0.431 | Ref |  |  |
|  | 12-17 years | 85 (100.0%) | 53 (62.4%) | 32 (37.6%) |  | 0.71 [0.42 ; 1.21] |  |  |
|  | Adults | 185 (100.0%) | 102 (55.1%) | 83 (44.9%) |  | 0.96 [0.63 ; 1.46] |  |  |
| **Smoking** | Missing | 1 | 1 | 0 |  |  |  |  |
|  | No | 413 (100.0%) | 227 (55.0%) | 186 (45.0%) | **0.073** * | Ref |  |  |
|  | Yes | 29 (100.0%) | 21 (72.4%) | 8 (27.6%) |  | 0.46 [0.20 ; 1.07] |  |  |
| **Use of cannabis** | Missing | 2 | 2 | 0 |  |  |  |  |
|  | No | 437 (100.0%) | 244 (55.8%) | 193 (44.2%) | 0.456 | Ref |  |  |
|  | Yes | 4 (100.0%) | 3 (75.0%) | 1 (25.0%) |  | 0.42 [0.04 ; 4.09] |  |  |
| **Conjunctivitis** | No | 193 (100.0%) | 120 (62.2%) | 73 (37.8%) | **0.026** * | Ref |  |  |
|  | Yes | 250 (100.0%) | 129 (51.6%) | 121 (48.4%) |  | 1.54 [1.05 ; 2.26] |  |  |
| **Sinusitis** | No | 294 (100.0%) | 181 (61.6%) | 113 (38.4%) | **0.002** * | Ref |  |  |
|  | Yes | 149 (100.0%) | 68 (45.6%) | 81 (54.4%) |  | 1.90 [1.28 ; 2.84] |  |  |
| **Atopic eczema** | No | 333 (100.0%) | 203 (61.0%) | 130 (39.0%) | **<0.001** * | Ref | **0.001** | Ref |
|  | Yes | 110 (100.0%) | 46 (41.8%) | 64 (58.2%) |  | 2.17 [1.40 ; 3.37] |  | 2.63 [1.45 ; 4.76] |
| **Food allergy** | No | 392 (100.0%) | 218 (55.6%) | 174 (44.4%) | 0.484 | Ref |  |  |
|  | Yes | 51 (100.0%) | 31 (60.8%) | 20 (39.2%) |  | 0.81 [0.44 ; 1.47] |  |  |
| **Allergic urticaria** | No | 399 (100.0%) | 224 (56.1%) | 175 (43.9%) | 0.931 | Ref |  |  |
|  | Yes | 44 (100.0%) | 25 (56.8%) | 19 (43.2%) |  | 0.97 [0.52 ; 1.82] |  |  |
| **Age of AR diagnosis** | Missing | 6 | 3 | 3 |  |  |  |  |
|  | <=1 years | 230 (100.0%) | 130 (56.5%) | 100 (43.5%) | 0.919 | Ref |  |  |
|  | >1 years | 207 (100.0%) | 116 (56.0%) | 91 (44.0%) |  | 1.020 [0.69 ; 1.49] |  |  |
| **Polysensitisation (Y/N)** | Missing | 5 | 2 | 3 |  |  |  |  |
|  | No | 166 (100.0%) | 95 (57.2%) | 71 (42.8%) | 0.783 | Ref |  |  |
|  | Yes | 272 (100.0%) | 152 (55.9%) | 120 (44.1%) |  | 1.06 [0.71 ; 1.56] |  |  |
| **Food allergy** | Missing | 5 | 2 | 3 |  |  |  |  |
|  | No | 397 (100.0%) | 219 (55.2%) | 178 (44.8%) | **0.110** * | Ref |  |  |
|  | Yes | 41 (100.0%) | 28 (68.3%) | 13 (31.7%) |  | 0.57 [0.29 ; 1.14] |  |  |
| **Allergy to Hymenoptera venoms** | Missing | 75 | 45 | 30 |  |  |  |  |
|  | No | 367 (100.0%) | 203 (55.3%) | 164 (44.7%) | NA |  |  |  |
|  | Yes | 1 (100.0%) | 1 (100.0%) | 0 (0.0%) |  |  |  |  |
| **Drug allergy** | Missing | 78 | 48 | 30 |  |  |  |  |
|  | No | 355 (100.0%) | 196 (55.2%) | 159 (44.8%) | 0.744 | Ref |  |  |
|  | Yes | 10 (100.0%) | 5 (50.0%) | 5 (50.0%) |  | 1.23 [0.35 ; 4.33] |  |  |
| **Occupational allergy** | Missing | 78 | 47 | 31 |  |  |  |  |
|  | No | 363 (100.0%) | 201 (55.4%) | 162 (44.6%) | 0.878 | Ref |  |  |
|  | Yes | 2 (100.0%) | 1 (50.0%) | 1 (50.0%) |  | 1.24 [0.08 ; 20.02] |  |  |
| **ARIA classification** | Missing | 28 | 11 | 17 |  |  |  |  |
|  | Mild persistent rhinitis | 81 (100.0%) | 51 (63.0%) | 30 (37.0%) | **0.121** * | Ref |  |  |
|  | Mild intermittent rhinitis | 53 (100.0%) | 36 (67.9%) | 17 (32.1%) |  | 0.80 [0.39 ; 1.67] |  |  |
|  | Severe persistent rhinitis | 274 (100.0%) | 146 (53.3%) | 128 (46.7%) |  | 1.49 [0.89 ; 2.48] |  |  |
|  | Severe intermittent rhinitis | 7 (100.0%) | 5 (71.4%) | 2 (28.6%) |  | 0.68 [0.12 ; 3.73] |  |  |
| **Sneezing** | No | 23 (100.0%) | 12 (52.2%) | 11 (47.8%) | 0.689 | Ref |  |  |
|  | Yes | 420 (100.0%) | 237 (56.4%) | 183 (43.6%) |  | 0.84 [0.36 ; 1.95] |  |  |
| **Rhinorrhoea** | No | 13 (100.0%) | 9 (69.2%) | 4 (30.8%) | 0.343 | Ref |  |  |
|  | Yes | 430 (100.0%) | 240 (55.8%) | 190 (44.2%) |  | 1.78 [0.54 ; 5.87] |  |  |
| **Nasal obstruction** | No | 31 (100.0%) | 14 (45.2%) | 17 (54.8%) | 0.202 | Ref |  |  |
|  | Yes | 412 (100.0%) | 235 (57.0%) | 177 (42.0%) |  | 0.62 [0.30 ; 1.29] |  |  |
| **Nasal pruritus** | No | 94 (100.0%) | 53 (56.4%) | 41 (43.6%) | 0.969 | Ref |  |  |
|  | Yes | 349 (100.0%) | 196 (56.2%) | 153 (43.8%) |  | 1.09 [0.64 ; 1.59] |  |  |
| **Loss of smell** | No | 239 (100.0%) | 152 (63.6%) | 87 (36.4%) | **<0.001** * | Ref | **0.008** | Ref |
|  | Yes | 204 (100.0%) | 97 (47.5%) | 107 (52.5%) |  | 1.93 [1.32 ; 2.82] |  | 2.03 [1.20 ; 3.43] |
| **Ocular pruritus** | No | 180 (100.0%) | 112 (62.2%) | 68 (37.8%) | **0.035** * | Ref |  |  |
|  | Yes | 263 (100.0%) | 137 (52.1%) | 126 (47.9%) |  | 1.52 [1.03 ; 2.23] |  |  |
| **Redness and tearing** | No | 202 (100.0%) | 128 (63.4%) | 74 (36.6%) | **0.006** * | Ref |  |  |
|  | Yes | 241 (100.0%) | 121 (50.2%) | 120 (49.8%) |  | 1.71 [1.17 ; 2.51] |  |  |
| **Control** | Missing | 80 | 51 | 29 |  |  |  |  |
|  | Well controlled | 214 (100.0%) | 145 (67.8%) | 69 (32.2%) | **<0.001** * | Ref | **0.002** | Ref |
|  | Partially controlled | 94 (100.0%) | 38 (40.4%) | 56 (59.6%) |  | 3.10 [1.87 ; 5.12] |  | 2.36 [1.29 ; 4.28] |
|  | Poorly controlled | 55 (100.0%) | 15 (27.3%) | 40 (72.7%) |  | 5.60 [2.90 ; 10.83] |  | 3.22 [1.43 ; 7.23] |
| **Current level of therapeutic load** | Missing | 6 | 6 | 0 |  |  |  |  |
|  | Level 1 | 167 (100.0%) | 118 (70.7%) | 49 (29.3%) | **<0.001** * | Ref | **0.012** | Ref |
|  | Level 2 | 102 (100.0%) | 54 (52.9%) | 48 (47.1%) |  | 2.14 [1.28 ; 3.57] |  | 2.41 [1.21 ; 4.81] |
|  | Level 3 | 128 (100.0%) | 56 (43.8%) | 72 (56.3%) |  | 3.10 [1.91 ; 5.02] |  | 2.75 [1.38 ; 5.46] |
|  | Level 4-5 | 40 (100.0%) | 15 (37.5%) | 25 (62.5%) |  | 4.01 [1.95 ; 8.26] |  | 2.89 [1.14 ; 7.34] |
| **NOSE score (class 1)** | Missing | 55 | 29 | 26 |  |  |  |  |
|  | <=50 | 177 (100.0%) | 106 (59.9%) | 71 (40.1%) | 0.246 | Ref |  |  |
|  | >50 | 211 (100.0%) | 114 (54.0%) | 97 (46.0%) |  | 1.27 [0.85 ; 1.90] |  |  |
| **RHINOQOL - Frequency** | Missing | 79 | 42 | 37 |  |  |  |  |
|  | >50 | 249 (100.0%) | 145 (58.2%) | 104 (41.8%) | 0.439 | Ref |  |  |
|  | <=50 | 115 (100.0%) | 62 (53.9%) | 53 (46.1%) |  | 1.19 [0.76 ; 1.86] |  |  |
| **RHINOQOL - Embarrassment** | Missing | 105 | 56 | 49 |  |  |  |  |
|  | >50 | 244 (100.0%) | 144 (59.0%) | 100 (41.0%) | 0.252 | Ref |  |  |
|  | <=50 | 94 (100.0%) | 49 (52.1%) | 45 (47.9%) |  | 1.32 [0.82 ; 2.13] |  |  |
| **RHINOQOL - Impact** | Missing | 69 | 36 | 33 |  |  |  |  |
|  | <=50 | 332 (100.0%) | 197 (59.3%) | 135 (40.7%) | **0.010** * | Ref |  |  |
|  | >50 | 42 (100.0%) | 16 (38.1%) | 26 (61.9%) |  | 2.371 [1.23 ; 4.59] |  |  |
| ** All variables with a p-value univariate <= 0.20 and with less than 20% missing data were selected for multivariate analysis ** "Stepwise" method with entry threshold at 0.20% and exit threshold at 0.05%* | | | | | | | | |

| Table 43 Asthmatic patient |
| --- |

|  | | **Total N=1701** |
| --- | --- | --- |
| **Asthmatic patient (Y/N)** | N | 1661 |
|  | Missing | 39 |
|  | No | 1089 (65.6%) |
|  | Yes | 572 (34.4%) |
|  | | |

|  | | **from 5 to 11 years N=513** | **12-17 years N=298** | **Adults N=875** |
| --- | --- | --- | --- | --- |
| **Asthmatic patient (Y/N)** | N | 505 | 288 | 854 |
|  | Missing | 8 | 10 | 20 |
|  | No | 290 (57.4%) | 182 (63.2%) | 606 (71.0%) |
|  | Yes | 215 (42.6%) | 106 (36.8%) | 248 (29.0%) |
|  | | | | |

| Table 44 If asthmatic patient, age of diagnosis of asthma (years) |
| --- |

|  | | **Total N=572** |
| --- | --- | --- |
| **Age of diagnosis of asthma (years)** | N | 544 |
|  | Missing | 28 |
|  | Mean ± ET | 5.2 ± 8.1 |
|  | Median | 2 |
|  | Q1 ; Q3 | 1 ; 6 |
|  | Min. ; Max. | 0 ; 63 |
|  | | |

|  | | **from 5 to 11 years N=215** | **12-17 years N=106** | **Adults N=248** |
| --- | --- | --- | --- | --- |
| **Age of diagnosis of asthma (years)** | N | 207 | 103 | 231 |
|  | Missing | 8 | 3 | 17 |
|  | Mean ± ET | 2.6 ± 2.5 | 3.4 ± 4.0 | 8.4 ± 11.1 |
|  | Median | 2 | 1 | 3 |
|  | Q1 ; Q3 | 1 ; 4 | 0 ; 6 | 1 ; 13 |
|  | Min. ; Max. | 0 ; 11 | 0 ; 15 | 0 ; 63 |
|  | | | | |

| Table 65 If asthmatic patient, Current level of therapeutic load (GINA 2017) |
| --- |

|  | | **Total N=572** |
| --- | --- | --- |
| **Current level of therapeutic load** | N | 563 |
|  | Missing | 9 |
|  | Level 1 | 231 (41.0%) |
|  | Level 2 | 133 (23.6%) |
|  | Level 3 | 154 (27.4%) |
|  | Level 4 | 44 (7.8%) |
|  | Level 5 | 1 (0.2%) |
|  | | |

|  | | **from 5 to 11 years N=215** | **12-17 years N=106** | **Adults N=248** |
| --- | --- | --- | --- | --- |
| **Current level of therapeutic load** | N | 213 | 105 | 242 |
|  | Missing | 2 | 1 | 6 |
|  | Level 1 | 86 (40.4%) | 46 (43.8%) | 99 (40.9%) |
|  | Level 2 | 64 (30.0%) | 20 (19.0%) | 46 (19.0%) |
|  | Level 3 | 56 (26.3%) | 27 (25.7%) | 71 (29.3%) |
|  | Level 4 | 6 (2.8%) | 12 (11.4%) | 26 (10.7%) |
|  | Level 5 | 1 (0.5%) | 0 (0.0%) | 0 (0.0%) |
|  | | | | |

| Table 53 If asthmatic patient, classification GINA |
| --- |

|  | | **Total N=572** |
| --- | --- | --- |
| **Control** | N | 468 |
|  | Missing | 104 |
|  | Well controlled | 274 (58.5%) |
|  | Partially controlled | 125 (26.7%) |
|  | Poorly controlled | 69 (14.7%) |
|  | | |

|  | | **from 5 to 11 years N=215** | **12-17 years N=106** | **Adults N=248** |
| --- | --- | --- | --- | --- |
| **Control** | N | 175 | 85 | 205 |
|  | Missing | 40 | 21 | 43 |
|  | Well controlled | 115 (65.7%) | 45 (52.9%) | 112 (54.6%) |
|  | Partially controlled | 43 (24.6%) | 23 (27.1%) | 58 (28.3%) |
|  | Poorly controlled | 17 (9.7%) | 17 (20.0%) | 35 (17.1%) |
|  | | | | |

| Table 93 If asthmatic patient (Adult or child 12 years of age or older), ACQ score in class |
| --- |

|  | | **Total N=303** |
| --- | --- | --- |
| **ACQ score in class** | N | 285 |
|  | Missing | 18 |
|  | <1 | 108 (37.9%) |
|  | 1-1.5 | 64 (22.5%) |
|  | >1.5 | 113 (39.6%) |
|  | | |

|  | | **12-17 years N=91** | **Adults N=212** |
| --- | --- | --- | --- |
| **ACQ score in class** | N | 84 | 201 |
|  | Missing | 7 | 11 |
|  | <1 | 39 (46.4%) | 69 (34.3%) |
|  | 1-1.5 | 17 (20.2%) | 47 (23.4%) |
|  | >1.5 | 28 (33.3%) | 85 (42.3%) |
|  | | | |

| Table 63* If asthmatic patient, number of severe or non-severe asthma exacerbations due to asthma in the past 12 months(ongoing) |
| --- |

** aberrant numbers of severe No exacerbations (>50) were considered "missing".*

|  | | **Total N=572** |
| --- | --- | --- |
| **Number of severe or non-severe asthma exacerbations in the past 12 months** | N | 443 |
|  | Missing | 129 |
|  | Mean ± ET | 2.4 ± 4.8 |
|  | Median | 1 |
|  | Q1 ; Q3 | 0 ; 3 |
|  | Min. ; Max. | 0 ; 54 |
|  | | |

|  | | **from 5 to 11 years N=215** | **12-17 years N=106** | **Adults N=248** |
| --- | --- | --- | --- | --- |
| **Number of severe or non-severe asthma exacerbations in the past 12 months** | N | 170 | 85 | 185 |
|  | Missing | 45 | 21 | 63 |
|  | Mean ± ET | 2.1 ± 2.9 | 2.8 ± 7.2 | 2.5 ± 4.9 |
|  | Median | 1 | 1 | 1 |
|  | Q1 ; Q3 | 0 ; 3 | 0 ; 2 | 0 ; 3 |
|  | Min. ; Max. | 0 ; 23 | 0 ; 54 | 0 ; 50 |
|  | | | | |

| Table 62* If asthmatic patient, number of severe or non-severe asthma exacerbations due to asthma in the past 12 months |
| --- |

** aberrant numbers of severe No exacerbations (>50) were considered "missing".*

|  | | **Total N=572** |
| --- | --- | --- |
| **Number of severe or non-severe asthma exacerbations in the past 12 months** | N | 443 |
|  |  |  |
|  | Missing | 129 |
|  | 0 | 181 (40.9%) |
|  | 1 | 68 (15.3%) |
|  | 2 | 57 (12.9%) |
|  | 3 | 50 (11.3%) |
|  | 4 | 28 (6.3%) |
|  | 5 | 12 (2.7%) |
|  | 6 | 14 (3.2%) |
|  | 7 | 4 (0.9%) |
|  | 8 | 3 (0.7%) |
|  | 9 | 3 (0.7%) |
|  | 10 | 10 (2.3%) |
|  | 11 | 1 (0.2%) |
|  | 12 | 1 (0.2%) |
|  | 13 | 1 (0.2%) |
|  | 15 | 3 (0.7%) |
|  | 18 | 1 (0.2%) |
|  | 20 | 2 (0.5%) |
|  | 23 | 1 (0.2%) |
|  | 30 | 1 (0.2%) |
|  | 50 | 1 (0.2%) |
|  | 54 | 1 (0.2%) |
|  | | |

| Table 105 If asthmatic patient, Number of severe asthma exacerbations in the past 12 months (ongoing) |
| --- |

|  | | **Total N=493** |
| --- | --- | --- |
| **Number of severe asthma exacerbations in the last 12 months** | N | 445 |
|  | Missing | 48 |
|  | Mean ± ET | 0.5 ± 1.2 |
|  | Median | 0 |
|  | Q1 ; Q3 | 0 ; 1 |
|  | Min. ; Max. | 0 ; 12 |
|  | | |

|  | | **from 5 to 11 years N=188** | **12-17 years N=91** | **Adults N=212** |
| --- | --- | --- | --- | --- |
| **Number of severe asthma exacerbations in the last 12 months** | N | 172 | 84 | 187 |
|  | Missing | 16 | 7 | 25 |
|  | Mean ± ET | 0.5 ± 1.0 | 0.5 ± 1.3 | 0.5 ± 1.4 |
|  | Median | 0 | 0 | 0 |
|  | Q1 ; Q3 | 0 ; 1 | 0 ; 0 | 0 ; 0 |
|  | Min. ; Max. | 0 ; 5 | 0 ; 10 | 0 ; 12 |
|  | | | | |

| Table 57 If asthmatic patient, oral corticosteroids >= 3 days, Asthma-related hospitalization more than 12 hours in the year |
| --- |

|  | | **Total N=572** |
| --- | --- | --- |
| **Taking oral corticosteroids >= 3 days** | N | 436 |
|  | Missing | 136 |
|  | No | 322 (73.9%) |
|  | Yes | 114 (26.1%) |
|  | | |
| **Asthma-related hospitalization more than 12 hours in the year** | N | 433 |
|  | Missing | 139 |
|  | No | 417 (96.3%) |
|  | Yes | 16 (3.7%) |
|  | | |

|  | | **from 5 to 11 years N=215** | **12-17 years N=106** | **Adults N=248** |
| --- | --- | --- | --- | --- |
| **Taking oral corticosteroids >= 3 days** | N | 169 | 79 | 188 |
|  | Missing | 46 | 27 | 60 |
|  | No | 116 (68.6%) | 62 (78.5%) | 144 (76.6%) |
|  | Yes | 53 (31.4%) | 17 (21.5%) | 44 (23.4%) |
|  | | | | |
| **Asthma-related hospitalization more than 12 hours in the year** | N | 168 | 79 | 186 |
|  | Missing | 47 | 27 | 62 |
|  | No | 159 (94.6%) | 77 (97.5%) | 181 (97.3%) |
|  | Yes | 9 (5.4%) | 2 (2.5%) | 5 (2.7%) |
|  | | | | |

**Table V :**

| Table 126* Number of exacerbations in the past 12 months(ongoing) |
| --- |

** Outliers of exacerbations (>50) were considered "missing".*

|  | | **Well controlled N=238** | **Partially controlled N=106** | **Poorly controlled N=65** | **P-value** |
| --- | --- | --- | --- | --- | --- |
| **How many times in the past 12 months** | N | 192 | 83 | 54 |  |
|  | Missing | 46 | 23 | 11 |  |
|  | Mean ± ET | 2.3 ± 2.3 | 2.4 ± 2.4 | 5.2 ± 9.8 |  |
|  | Median | 2 | 2 | 3 | **0.0422** [f] |
|  | Min. ; Max. | 0 ; 10 | 0 ; 12 | 0 ; 50 |  |
|  | | | | | |
| *[f]: Kruskal-Wallis test* | | | | | |

| Table 116 Age of diagnosis of mite rhinitis(years) |
| --- |

|  | | **Well controlled N=274** | **Partially controlled N=125** | **Poorly controlled N=69** |
| --- | --- | --- | --- | --- |
| **Age of diagnosis of mite rhinitis (years)** | N | 270 | 125 | 68 |
|  | Missing | 4 | 0 | 1 |
|  | Mean ± ET | 3.1 ± 5.3 | 4.4 ± 7.6 | 4.6 ± 6.9 |
|  | Median | 1 | 1 | 2 |
|  | Q1 ; Q3 | 0 ; 3 | 0 ; 6 | 0 ; 6 |
|  | Min. ; Max. | 0 ; 33 | 0 ; 57 | 0 ; 37 |
|  | | | | |

| Table 127 Symptoms questionnaire NOSE |
| --- |

|  | | **Well controlled N=238** | **Partially controlled N=106** | **Poorly controlled N=65** | **P-value** |
| --- | --- | --- | --- | --- | --- |
| **NOSE score (0-100)** | N | 236 | 106 | 65 |  |
|  | Missing | 2 | 0 | 0 |  |
|  | Mean ± ET | 49.9 ± 26.5 | 57.1 ± 24.4 | 58.2 ± 28.8 |  |
|  | Median | 50 | 60 | 60 | **0.009** [f] |
|  | Q1 ; Q3 | 30 ; 70 | 40 ; 75 | 40 ; 80 |  |
|  | Min. ; Max. | 0 ; 100 | 0 ; 100 | 0 ; 100 |  |
|  | | | | | |
| *[f]:  Kruskal-Wallis test* | | | | | |

| 0=Patients without symptoms, 100=Patients with the most intense symptoms possible |
| --- |

| Table 128 NOSE score >50 | | | | | |
| --- | --- | --- | --- | --- | --- |
|  | | **Well controlled N=238** | **Partially controlled N=106** | **Poorly controlled N=65** |  |
| **NOSE score (class 1)** | N | 236 | 106 | 65 |  |
|  | Missing | 2 | 0 | 0 |  |
|  | >50 | 110 (46.6%) | 63 (59.4%) | 38 (58.5%) |  |
|  | <=50 | 126 (53.4%) | 43 (40.6%) | 27 (41.5%) |  |
|  | | | | |  |

| Table 129 RHINOQOL |
| --- |

|  | | **Well controlled N=238** | **Partially controlled N=106** | **Poorly controlled N=65** | **P-value** |
| --- | --- | --- | --- | --- | --- |
| **RHINOQOL - Frequency (0-100)** | N | 224 | 98 | 61 |  |
|  | Missing | 14 | 8 | 4 |  |
|  | Mean ± ET | 64.4 ± 21.0 | 59.9 ± 18.4 | 62.5 ± 25.5 |  |
|  | Median | 63 | 63 | 69 | 0.191 [f] |
|  | Q1 ; Q3 | 50 ; 81 | 44 ; 75 | 44 ; 81 |  |
|  | Min. ; Max. | 13 ; 100 | 13 ; 100 | 0 ; 100 |  |
|  | | | | | |
| **RHINOQOL - Embarrassment (0-100)** | N | 207 | 93 | 56 |  |
|  | Missing | 31 | 13 | 9 |  |
|  | Mean ± ET | 66.2 ± 22.9 | 62.9 ± 20.3 | 67.4 ± 22.4 |  |
|  | Median | 67 | 60 | 70 | 0.284 [f] |
|  | Q1 ; Q3 | 50 ; 83 | 47 ; 77 | 47 ; 87 |  |
|  | Min. ; Max. | 0 ; 100 | 23 ; 100 | 20 ; 100 |  |
|  | | | | | |
| **RHINOQOL - Impact (0-100)** | N | 231 | 104 | 59 |  |
|  | Missing | 7 | 2 | 6 |  |
|  | Mean ± ET | 23.1 ± 20.3 | 28.0 ± 20.2 | 29.8 ± 23.5 |  |
|  | Median | 19 | 28 | 25 | **0.026** [f] |
|  | Q1 ; Q3 | 6 ; 36 | 11 ; 43 | 6 ; 50 |  |
|  | Min. ; Max. | 0 ; 83 | 0 ; 89 | 0 ; 100 |  |
|  | | | | | |
| *[f]:  Kruskal-Wallis test* | | | | | |

| RhinoQOL score Frequency (0-100) : The higher the score, the less frequent the symptoms are |
| --- |
| RhinoQOL score Embarrassment (0-100) : the higher the score, the less discomfort is |
| RhinoQOL score Impact (0-100) : the higher the score, the more pejorative the impact on quality of life is |

| Table 131 DYNACHRON |
| --- |

|  | | **Well controlled N=238** | **Partially controlled N=106** | **Poorly controlled N=65** |
| --- | --- | --- | --- | --- |
| **Partial DYNACHRON score (0-130)** | N | 229 | 101 | 61 |
|  | Missing | 9 | 5 | 4 |
|  | Mean ± ET | 44.8 ± 31.7 | 51.9 ± 29.5 | 55.9 ± 33.4 |
|  | Median | 41 | 59 | 57 |
|  | Q1 ; Q3 | 19 ; 69 | 26 ; 72 | 33 ; 83 |
|  | Min. ; Max. | 0 ; 130 | 0 ; 115 | 0 ; 130 |
|  | | | | |

| DYNACHRON score (0-130) : the higher the score, the greater the embarrassment is |
| --- |

| Table 132 If asthmatic patient (Adult or child 12 years of age or older), ACQ score | | | | | | |
| --- | --- | --- | --- | --- | --- | --- |
|  | | **Well controlled N=134** | **Partially controlled N=70** | **Poorly controlled N=49** | **P-value** |  |
| **ACQ score** | N | 124 | 67 | 48 |  |  |
|  | Missing | 10 | 3 | 1 |  |  |
|  | Mean ± ET | 1.0 ± 0.9 | 1.6 ± 1.0 | 2.4 ± 1.2 |  |  |
|  | Median | 1 | 2 | 3 | **<0.001** [f] |  |
|  | Q1 ; Q3 | 0 ; 2 | 1 ; 3 | 2 ; 3 |  |  |
|  | Min. ; Max. | 0 ; 6 | 0 ; 4 | 0 ; 5 |  |  |
|  | | | | | |  |
| *[f]: Kruskal-Wallis test* | | | | | |  |

| Means of 6 items (from 0-6, score 6 is the worst) |
| --- |

| Table 99* Average duration of symptoms (days) - In patients with at least one exacerbation |
| --- |

** Outliers of exacerbations (>300) were considered "missing".*

|  | | **Total N=906** |
| --- | --- | --- |
| **Average duration of symptoms (days)** | N | 834 |
|  | Missing | 72 |
|  | Mean ± ET | 14.1 ± 17.1 |
|  | Median | 8.67 |
|  | Min. ; Max. | 1 ; 185 |
|  | | |

|  | | **from 5 to 11 years N=250** | **12-17 years N=149** | **Adults N=498** |
| --- | --- | --- | --- | --- |
| **Average duration of symptoms (days)** | N | 235 | 130 | 461 |
|  | Missing | 15 | 19 | 37 |
|  | Mean ± ET | 12.9 ± 16.2 | 13.4 ± 15.1 | 14.8 ± 18.1 |
|  | Median | 8.33 | 8.00 | 10.00 |
|  | Min. ; Max. | 1 ; 180 | 1 ; 105 | 1 ; 185 |
|  | | | | |

**Figure I :**

Figure 1 : Flow chart of study populations

**Physician population**

N = 151 physicians

N = 195 médecins

Number of patients per physician

mean : 11,4

Total population

N = 1725 patients

Population totale

N = 1600 patients

At least one major deviation from the protocol : **24 patients (1,4%)**

**Patient population included**

N = 1701 patients

N = 1589 patients

**12-17 years**

N= 298 patients (17,7%)

**5-11 years**

N= 513 patients (30,4%)

**Adults**

N= 875 patients (51,9%)

**Patient population Self-questionnaires**

N=1486 (86,1%)

**Figure II :**

**Adults**

N= 771 patients (51,9%)

**5-11 years**

N= 439 patients (29,5%)

**12-17 years**

N= 263 patients (17,7%)

#### Table 97b* Month in the year - In patients with at least 2 exacerbations

|  | **Total (N=636)** | |  |
| --- | --- | --- | --- |
|  | **n (1)** | **% (2)** |  |
| TOTAL | 581 | 91.4 |  |
| OCTOBER | 252 | 39.6 |  |
| SEPTEMBER | 250 | 39.3 |  |
| NOVEMBER | 219 | 34.4 |  |
| DECEMBER | 183 | 28.8 |  |
| AUGUST | 128 | 20.1 |  |
| JANUARY | 124 | 19.5 |  |
| JULY | 111 | 17.5 |  |
| MARCH | 107 | 16.8 |  |
| FEBRUARY | 102 | 16.0 |  |
| JUNE | 91 | 14.3 |  |
| APRIL | 92 | 14.5 |  |
| MAY | 85 | 13.4 |  |
|  | | | |
| (1) Number of patients with at least one symptom | | | |
| (2) (n/N)*100 (N: Number of patients) | | | |
